# Matrons, and Nurse Agitators

**Published:** 1918-09-21

**Authors:** 


*To the Editor of* THE HOSPITAL.

### Matrons, and Nurse Agitators.

*To the Editor of* THE HOSPITAL.

SIR,—As neither a matron nor a third-year probationer may I suggest that the nurse agitator call a meeting of the various matrons and the 5,000 nurses in training, and, taking the platform herself, voice her grievances? Who was it said Hyde Park is the Englishman's "safety valve"? Doubtless in such a good cause the hospital patients would look after themselves *pro tem.* Probably some of the 4,999 would still be too busy nursing the sick to attend, but those agitators who are leading a "dog's life" would have an opportunity of expressing their dissatisfaction with the manner in which the matrons carry out their duties. It seems a comparatively trivial thing that "Third Year Pro." cannot obtain anything higher than a "good" certificate, since she appears to have such unique opportunities of judging which of her fellow-nurses are the good, conscientious workers and administrators of the future, so early in her career. Her sister and matron are probably working at high pressure. The thorny path of a matron should soon be hers. Let her look to it that she be one of those who will truly be a friend and adviser, and from a multitude of duties be ever ready to receive a deputation of "nurse agitators." It is certain there will always be some who find it impossible under any circumstances to get on without a grievance.

A TEN YEARS' NURSE.

September 14.

